# Anthropometric Analysis of the Clinically Measured Breast Footprint: An Exploratory Study of BMI and Thoracic Width Associations with Apparent Horizontal Expansion

**DOI:** 10.3390/jcm15052028

**Published:** 2026-03-06

**Authors:** Ibrahim Güler, Gerrit Grieb, Armin Kraus, Henrik Stelling

**Affiliations:** 1 Department of Plastic, Aesthetic and Hand Surgery, Otto-von-Guericke University, 39120 Magdeburg, Germany; armin.kraus@med.ovgu.de; 2 Department of Health Management, Friedrich-Alexander-Universität Erlangen-Nürnberg, Lange Gasse 20, 90403 Nürnberg, Germany; 3 Department of Plastic Surgery and Hand Surgery, Gemeinschaftskrankenhaus Havelhoehe, Kladower Damm 221, 14089 Berlin, Germany; gerritgrieb@gmx.de; 4 Department of Plastic Surgery and Hand Surgery, Burn Center, Medical Faculty, RWTH Aachen University, Pauwelsstrasse 30, 52074 Aachen, Germany; 5 Practices for Nuclear Medicine, Rubensstraße 125, 12157 Berlin, Germany; henrikstelling@googlemail.com

**Keywords:** breast footprint, breast anthropometry, body mass index, thoracic width, height-to-base-width ratio, breast base width, breast morphology, soft-tissue distribution, aesthetic breast surgery, surface-based measurements

## Abstract

**Background/Objectives**: Breast morphology is commonly described using volume, projection, and ptosis, whereas proportional relationships within the breast footprint are less frequently quantified. Routine clinical measurements describe the apparent or clinically measured breast footprint rather than the fixed anatomical footprint. This exploratory study examines how body mass index and thoracic width are associated with the clinically measured horizontal and vertical dimensions of the breast footprint and introduces the height-to-base-width (H/B) ratio as a simple descriptive index. **Methods**: Anthropometric measurements from 50 women undergoing aesthetic breast surgery were retrospectively analyzed. Breast base width and breast height were obtained using standardized upright clinical measurements. BMI was used as a surrogate of adiposity, while thoracic circumference measured at the inframammary fold (band size) served as a proxy for thoracic frame size. Associations were examined using Spearman correlation and multivariable regression. **Results**: BMI showed a strong positive association with clinically measured breast base width (ρ = 0.691, *p* < 0.001) but only a weak association with breast height (ρ = 0.327, *p* = 0.0777). Thoracic width was inversely associated with the H/B ratio (ρ = −0.549, *p* = 0.002). Multivariable analysis identified BMI as the principal determinant of measured base width, whereas vertical footprint dimensions showed limited responsiveness to BMI variation. **Conclusions**: Higher BMI was associated with horizontal expansion of the measured breast footprint, while vertical dimensions remained comparatively stable. These findings reflect soft-tissue redistribution and measurement-dependent footprint appearance rather than alteration of the underlying anatomical footprint. The H/B ratio emerges as a potential descriptive index for apparent footprint proportions, meriting further investigation and prospective validation.

## 1. Introduction

Breast morphology is commonly described using volumetric assessments, linear surface measurements, and ptosis classifications. These metrics supply useful clinical information but typically focus on size, ptosis and projection rather than proportional geometry. Prior anthropometric work has documented standard linear measures such as breast base width, suprasternal notch–to–nipple distance, nipple to inframammary fold distance and inter-nipple distance across diverse populations. Three-dimensional imaging studies have further characterized contour and projection although such approaches require volumetric data and specialized equipment. Clinical experience also suggests that body mass and the thoracic frame both influence breast appearance, yet quantitative data on how body mass index and thoracic width jointly shape the clinically measured relationship between vertical and horizontal dimensions remain limited [1,2,3,4,5,6,7,8,9].

Hall-Findlay conceptualized breast analysis in three dimensions, with the first two dimensions forming the “breast footprint” defined by its vertical and horizontal borders, and the third dimension describing the breast shape that sits upon this footprint. Although her framework emphasizes understanding which components of the footprint can and cannot be altered surgically, the present study focuses on the proportional geometry of the clinically assessed footprint, acknowledging that surface measurements reflect apparent dimensions rather than the fixed anatomical footprint defined by ligamentous attachments [10]. The introduced height-to-base-width (H/B) index therefore provides a simple numeric descriptor of this footprint geometry by expressing vertical height relative to horizontal base width.

**Figure 1:**
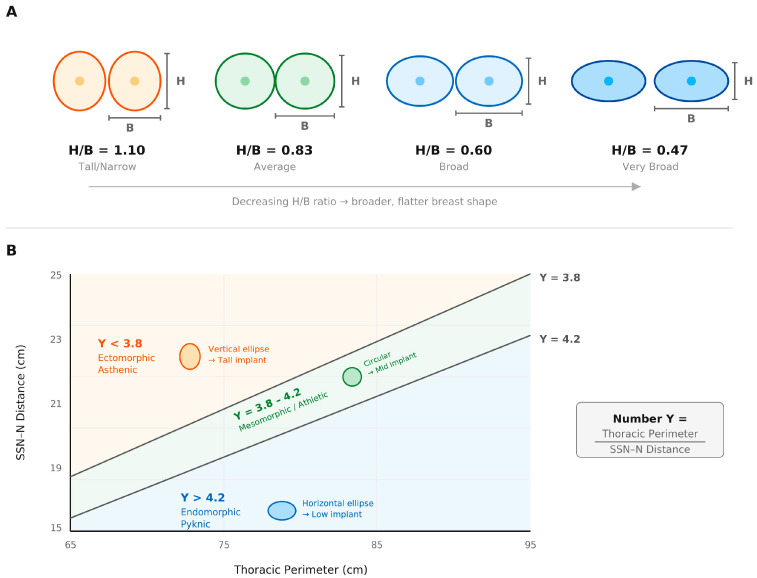
(**A**) is a conceptual illustration of the breast height-to-base-width (H/B) form index. Lower H/B values correspond to broader, flatter breast shapes, whereas higher values indicate taller and narrower proportions. (**B**) overlays the Del Yerro Number-Y zones onto a plot of thoracic perimeter (*x*-axis) versus suprasternal-notch-to-nipple (SSN–N) distance (*y*-axis). Number-Y (thoracic perimeter/SSN–N) categorizes non-ptotic breasts according to somatotype: ectomorphic (Y < 3.8) with a vertical-ellipse implantation base; mesomorphic (Y 3.8–4.2) with a circular base; and pyknic/endomorphic (Y > 4.2) with a horizontal-ellipse implantation base. These zones correspond to characteristic footprint orientations used in implant height selection. Thus, (**B**) illustrates how skeletal frame and vertical positioning (SSN–N distance) jointly define the inherent breast implantation base, complementing the footprint-proportion concept shown in (**A**). Number-Y is shown here as a related conceptual framework to contextualize footprint orientation; it is provided for overview only and was not applied in the analyses of the present study [11].

We therefore evaluate a simple form index derived from routine clinical surface measurements to describe apparent footprint proportions rather than intrinsic anatomical footprint characteristics and examine how this index relates to BMI and bra band size in a surgical cohort. A conceptual illustration of the H/B form index, demonstrating the relationship between vertical breast height and horizontal base width, is shown in Figure 1.

## 2. Materials and Methods

### 2.1. Study Population

We retrospectively analyzed anthropometric data from 50 consecutive female patients who underwent aesthetic breast surgery at our institution. Planned procedures included breast reduction or mastopexy, implant exchange, breast augmentation, and autologous lipofilling. All measurements were obtained during routine clinical care. No additional measurements were performed for this study. All data were irreversibly anonymized at the time of extraction and analyzed exclusively in aggregated form, with no personal or indirectly identifiable information.

### 2.2. Anthropometric Measurements

Standardized clinical measurements were performed with patients in upright standing position. Recorded metrics included:

- Suprasternal notch to nipple distance (SSN–N, bilateral, cm);
- Nipple to inframammary fold distance (N–IMF, bilateral, cm);
- Breast base width (horizontal span, bilateral, cm);
- Breast height (vertical span, bilateral, cm);
- Inter-nipple distance (IND, single midline measurement, cm);
- Thoracic circumference measured at the inframammary fold (cm), hereafter referred to as band size;
- Body mass index (BMI, kg/m^2^);
- Height (cm);
- Weight (kg).

Breast base width was defined as a horizontal linear distance, measured from the medial point where the breast parenchyma begins to rise from the chest wall to the most lateral palpable border of the breast parenchyma. Measurements were obtained with a vernier caliper in the standing position. The measurement was performed as a straight-line distance and not along the skin surface contour.

Breast height was defined as the vertical linear distance from the upper palpable border of the breast parenchyma (upper pole) to the inframammary fold.

For both parameters, particular care was taken in routine clinical practice to consistently identify the transition between breast tissue and surrounding soft tissues. This transition is inherently gradual and may be less clearly delineated in patients with higher body mass index, representing a known source of measurement variability.

BMI served as a measure of adiposity and general soft-tissue bulk, while band size was used as a pragmatic, clinically available approximation of thoracic frame size. As a soft-tissue–inclusive measure, band size does not represent a pure skeletal dimension but allows aspects of overall body size to be modeled separately in an exploratory framework.

Breast height in this study refers to the vertical dimension of the two-dimensional breast footprint, distinct from the three-dimensional mound projection. Consequently, this metric does not capture the ptosis-related elongation typically seen in the suprasternal notch–to-nipple distance. By differentiating the fixed anatomical base from the mobile soft-tissue envelope, we consider that age-related or ligament-related descent primarily affects the breast mound and is unlikely to be the dominant determinant of the height measurements analyzed here, although a residual influence cannot be fully excluded in a clinical, surface-based assessment.

Measurements were obtained during outpatient clinic visits by two board-certified plastic surgeons using a standard tape measure and a caliper. Operators followed a standardized protocol as recommended in prior anthropometric studies, which suggests that consistent use of a defined protocol can reduce measurement variability. Inter- and intra-operator reliability were not formally assessed and thus represent a methodological limitation, as discussed below [1,3].

Not all listed measurements were available for all patients because certain anthropometric variables were not consistently obtained during routine clinical care. Sample size therefore varies across variables (see Table 1). These missing values reflect incomplete routine documentation rather than study-related exclusion; however, non-random missingness cannot be fully ruled out. Given the retrospective design and limited sample size, all analyses involving variables with reduced n should be regarded as exploratory in nature.

Left and right values were averaged to obtain single representative metrics per patient. As the primary aim of this study was to model the scaling of breast footprint geometry with body habitus rather than to quantify asymmetry, this mean value provides the most accurate representation of the patient’s dominant phenotype. From these primary measurements we derived the form index H/B ratio, computed as breast height divided by base width.

All reported measurements represent surface-based, clinically obtained dimensions and do not directly assess the fixed skeletal or fascial boundaries of the anatomical breast footprint. The term ‘footprint’ is therefore used throughout this study to denote the clinically measured or apparent footprint.

### 2.3. Statistical Analysis

All statistical analyses were performed using Python 3.12 with the following libraries: pandas (v2.2), numpy (v1.26), scipy (v1.13), statsmodels (v0.14), and matplotlib (v3.8). Descriptive statistics are presented as mean ± standard deviation (SD) with 95% confidence intervals (CI) for the mean, calculated as mean ± 1.96 × standard error [12].

Bivariate associations were assessed using Spearman rank correlation coefficients (ρ) due to potential non-linearity and outliers. To address multiple testing, we applied the Benjamini–Hochberg false discovery rate (FDR) correction, reporting *q*-values alongside raw *p*-values. For transparency, we report both raw *p*-values and FDR-adjusted *q*-values; statistical interpretation is based primarily on the adjusted *q*-values [13,14,15].

Multivariable relationships were examined using ordinary least squares (OLS) regression [16]. To enable comparison of predictor strengths despite different measurement scales, we employed semi-standardized coefficients: predictors (BMI, band size) were standardized to their population standard deviation (mean = 0, SD = 1), while outcomes remained in original units. This approach yields coefficients interpretable as “change in outcome per 1-SD increase in predictor” while preserving clinically meaningful outcome scales. Regression model assumptions, including residual distribution and multicollinearity, were checked and no major violations were observed; multicollinearity diagnostics are reported in Section 3.3.

Statistical significance was defined as two-tailed *p* < 0.05 for regression models and FDR-adjusted *q* < 0.05 for bivariate correlations. For marginal findings (0.05 ≤ *p* < 0.10), we report exact *p*-values and discuss implications cautiously [17].

### 2.4. Artificial Intelligence

Generative AI–based tools (Claude Sonnet 4.5) were used during manuscript preparation to support the authors in auxiliary tasks, including language editing, grammar and stylistic refinement, as well as assistance with code generation.

## 3. Results

### 3.1. Sample Characteristics

The cohort consisted of 50 patients with a broad range of body and breast measurements (Table 1). All values originated from standardized upright clinical assessments. Primary recorded metrics included BMI, band size, suprasternal-to-nipple distance, nipple-to-IMF distance, breast base width, breast height, and inter-nipple distance. For variables measured bilaterally, left and right values were averaged to generate a single patient-level measurement. Not all parameters were available for all individuals, resulting in variable sample sizes across measurements. Breast base width and height were available in 32 and 30 patients respectively. Despite these variations in data completeness, the study population covered a broad spectrum of body habitus (BMI range 18.2–39.9 kg/m^2^), ensuring sufficient variability for correlation analysis. Planned surgical procedures included reduction or mastopexy in 52%, implant exchange in 32%, augmentation in 12%, and lipofilling in 4% of cases (Table 2).

### 3.2. Bivariate Associations

BMI demonstrated a moderate-to-strong positive correlation with breast base width and a weaker, non-significant correlation with breast height (Table 3). Thoracic width (band size) also correlated positively with base width and showed a significant inverse association with the H/B form index, whereas BMI showed a weaker association. By contrast, neither BMI nor band size showed a statistically significant association with breast height; in this sample, vertical breast dimension appeared relatively independent of overall body habitus. These bivariate patterns are summarized in Table 3.

### 3.3. Multivariable Models

While bivariate analyses demonstrated individual associations, multivariable regression was performed to disentangle the independent contributions of thoracic width versus adiposity, which are naturally correlated. In multivariable ordinary least squares regression, BMI remained an independent predictor of breast base width, whereas band size contributed a smaller but statistically significant additional effect (Table 4). For the H/B form index, band size showed a marginal inverse association after adjustment for BMI, while BMI showed no independent effect. Neither predictor explained meaningful variance in breast height in this sample, which exhibited considerable inter-individual variability.

## 4. Discussion

**Figure 2:**
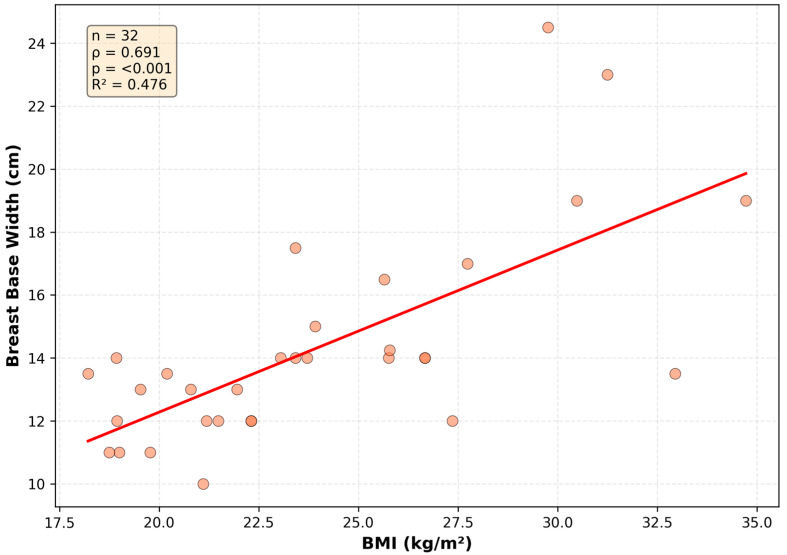
Association between BMI and breast base width. BMI shows a positive association with horizontal breast expansion. The data show a consistent positive correlation (n = 32, Spearman ρ = 0.691, *p* < 0.001). The linear fit illustrates the strength of the observed association (R^2^ = 0.476).

**Figure 3:**
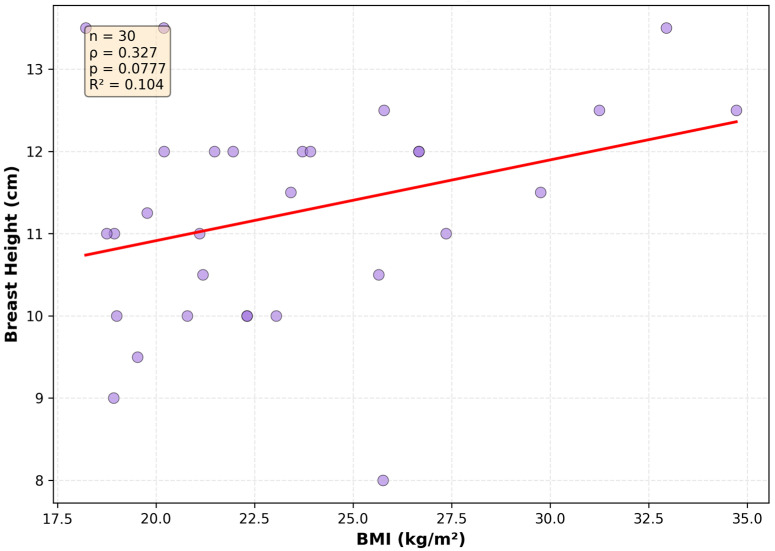
Relationship between BMI and breast height. Unlike base width, vertical breast height shows no statistically significant correlation with BMI (n = 30, Spearman ρ = 0.327, *p* = 0.0777). This suggests the vertical dimension remains relatively constrained by anatomical boundaries (R^2^ = 0.109).

This study provides preliminary quantitative evidence that horizontal and vertical breast dimensions may respond differently to variation in body habitus in a clinical cohort. While prior anthropometric work has described normative linear measurements across populations [1,2,3,4,5,6,7,8,9], most existing reports treat these dimensions as independent static values and rarely examine how they scale with body weight or thoracic anatomy. Our findings show a consistent pattern: BMI showed a significant association with breast base width (Figure 2; Spearman ρ = 0.69, R^2^ = 0.48) but showed only a weak and non-significant association with vertical breast height (Figure 3; Spearman ρ = 0.33, R^2^ = 0.10). This weak positive trend (*p* = 0.0777) may reflect insufficient statistical power rather than true absence of association. However, given the limited number of patients with complete height measurements, this interpretation should be viewed with caution.

An important consideration in interpreting these findings is that all reported dimensions refer to the clinically measured breast footprint. Several authors have argued that the anatomical breast footprint—defined by ligamentous attachments and skeletal boundaries—represents a relatively fixed entity that does not substantially change with weight variation [18,19]. The present study does not challenge this concept. Instead, it describes how breast dimensions appear when assessed using routine surface-based clinical measurements. The observed association between BMI and base width likely reflects lateral soft-tissue accumulation along the thoracic wall, which increases the measured horizontal extent without necessarily altering the underlying anatomical footprint. It should be noted that surgical indication may partially contribute to this association, as patients presenting for breast reduction often exhibit both higher BMI and inherently wider breast bases, whereas augmentation candidates tend to present with narrower baseline dimensions.

**Figure 4:**
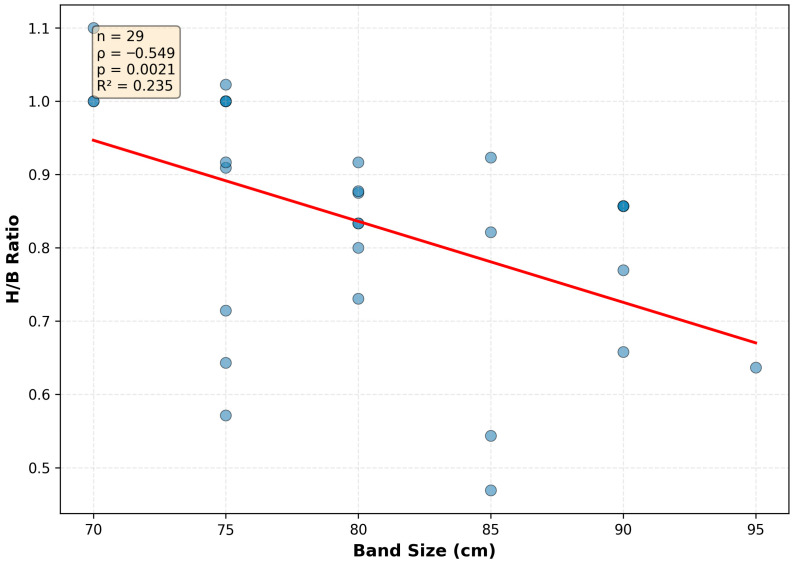
Correlation between thoracic width and H/B form index. The scatterplot demonstrates a significant inverse relationship, indicating that larger thoracic circumference is associated with flatter measured breast footprints (n = 29, Spearman ρ = −0.549, *p* = 0.0021). The red line represents the linear fit (R^2^ = 0.235).

**Figure 5:**
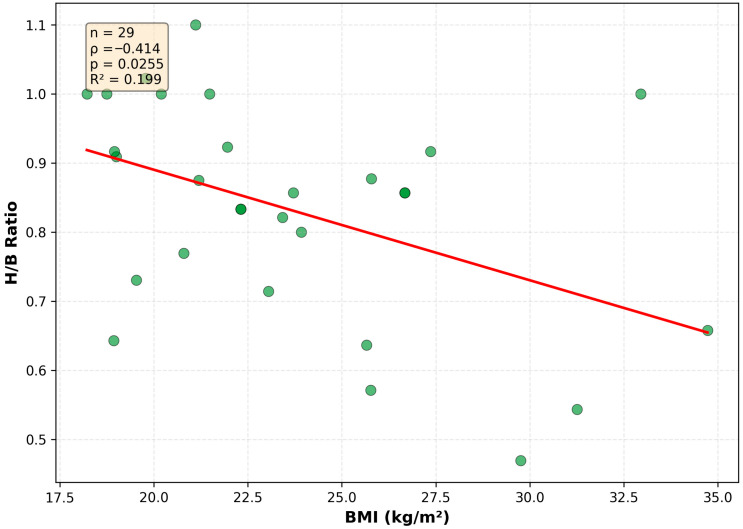
Impact of BMI on breast footprint proportions. Higher body mass index shows a moderate inverse association with the H/B ratio, reflecting a shift toward broader, flatter breast shapes (n = 29, Spearman ρ = −0.414, *p* = 0.0255). The red line indicates the linear trend (R^2^ = 0.199).

Thoracic width further modifies this relationship. Broader chests (larger band size) are significantly associated with flatter, more horizontally oriented measured breast proportions (Figure 4; Spearman ρ = −0.55, R^2^ = 0.24). BMI also shows an inverse association with the H/B ratio (Figure 5; Spearman ρ = −0.41, R^2^ = 0.20). Together, these findings indicate that the vertical footprint dimension shows relative stability across different body habitus profiles, whereas the horizontal dimension expands readily with increasing soft-tissue mass as reflected in surface-based measurements.

These findings are consistent with Hall-Findlay’s conceptual description of the vertical footprint as a dimension conceptually constrained by relatively fixed inferior and superior borders (inframammary fold inferiorly and the chest-wall/breast junction superiorly) [10]. Unlike measured horizontal width, which increases with soft-tissue accumulation, the vertical extent of the breast is likely anchored by these skeletal and fascial boundaries and may therefore show limited responsiveness to weight gain, although this interpretation remains preliminary given the clinical nature of the assessment and the limited sample size. This relative stability of breast height offers a plausible conceptual explanation for the declining H/B index with rising BMI: measured width increases, while height remains anatomically constrained. Although upper-pole fullness may fluctuate with soft-tissue changes, the superior boundary of the vertical footprint remains defined by the fixed chest-wall–breast junction. Thus, the observed stability of breast height in surface measurements is consistent with the concept of a stable anatomic footprint rather than transient soft-tissue variation [20].

Additional support for the relative stability of the vertical footprint comes from Martín del Yerro and Biggs, who introduced the Number-Y construct to describe somatotype-dependent variation in the breast implantation base [11]. Their model demonstrates that thoracic perimeter and SSN–N distance scale in predictable patterns across ectomorphic, mesomorphic and endomorphic body types, yet the orientation of the breast base (vertical vs. horizontal dominance) is conceptualized as largely anatomically predetermined. Ectomorphic patients (Y < 3.8) tend to show tall, narrow, vertically oriented bases, whereas endomorphic patients (Y > 4.2) exhibit broad, horizontally dominant bases—a pattern driven more by skeletal and soft-tissue distribution than by weight-related vertical elongation. This framework parallels our findings: measured horizontal dimensions vary substantially with habitus, while vertical height remains tightly constrained, reinforcing the value of simple proportional indices such as the H/B ratio for characterizing apparent footprint geometry.

Our results align with commonly reported clinical impressions. First, weight gain is often perceived to produce broader, flatter breast outlines rather than vertical elongation. The present analysis provides quantitative support for this impression and suggests that the effect is primarily driven by increases in measured base width. Second, because base width is influenced by thoracic size as assessed by routine clinical measurement, variation in thoracic circumference (band size) modulates the H/B index, with wider measured circumferences tending toward lower H/B values (broader, flatter apparent footprints). This pattern, soft-tissue mass expanding the apparent base while vertical dimensions remain relatively constrained, illustrates why simple proportional measures such as the H/B ratio may be useful for descriptive documentation of apparent footprint geometry in the context of weight variation.

### 4.1. Interpretation of Marginal Findings

The multivariable trend for band size predicting H/B narrowly missed conventional significance in our sample (*p* ≈ 0.05) but showed a consistent bivariate correlation. Given limited power for the H/B subset, we treat this result as suggestive and biologically plausible rather than definitive; larger cohorts are needed to confirm an independent skeletal effect beyond BMI.

### 4.2. Clinical Implications

- The H/B ratio is a simple, surface-based descriptive index that expresses vertical height relative to horizontal base width using routine clinical measurements. In its current form, it is intended for descriptive documentation and hypothesis generation rather than direct decision-making.
- These preliminary findings suggest that weight gain may preferentially widen the apparent footprint horizontally, though this requires prospective confirmation before informing surgical planning.
- Normalizing horizontal measurements such as inter-nipple distance to thoracic circumference may help distinguish frame-related variation from breast-specific findings, though this was not formally tested in the present study.
- Counseling point: patients should be informed that weight change may affect horizontal and vertical breast dimensions differently; the interplay between measured base width changes, skin envelope laxity, and tissue quality during weight fluctuation is complex and should be addressed individually.

### 4.3. Limitations

- Because only surface-based anthropometric measurements were available, conclusions are limited to apparent footprint dimensions and cannot directly address changes of the fixed anatomical footprint.
- The study cohort consisted exclusively of patients seeking aesthetic breast surgery, introducing selection bias toward individuals with specific morphological concerns. Findings may not be fully generalizable to the general female population.
- Retrospective, single-center design and modest sample size limit generalizability, particularly to populations with different ethnic backgrounds or body composition patterns, and reduce power for subgroup analysis (age, parity, prior weight change).
- Manual linear anthropometry (upright position) was employed for clinical applicability. While validated in prior anthropometric work, it does not capture full 3D contour or volumetric distribution. Three-dimensional imaging adds valuable detail on projection and lower-pole geometry but is less widely available and more costly.
- Band size (thoracic circumference) was used as a pragmatic surrogate for thoracic width. As a soft-tissue-inclusive measure, it imperfectly reflects bony chest dimensions. Direct skeletal measures or imaging would provide a cleaner assessment of thoracic contribution.
- Important covariates such as parity, breast density, hormonal status, prior pregnancies and breastfeeding, and time since weight change were not consistently available and could confound associations.
- Cross-sectional design precludes causal inference: observed patterns indicate association but not the intra-individual dynamics of shape change over time (weight gain versus weight loss).
- The H/B index describes apparent footprint geometry only; it does not capture mound projection, upper-pole fullness, or skin envelope quality, all of which meaningfully influence aesthetic outcome.
- The cohort included patients with diverse surgical indications (reduction, augmentation, mastopexy, revision), which represent morphologically distinct baseline conditions (e.g., macromastia and ptosis vs. hypomastia). Baseline breast morphology may differ systematically across these groups; stratified analyses were precluded by sample size but represent an important direction for larger studies.
- A substantial proportion of missing measurements resulted from retrospective documentation, and non-random missingness cannot be excluded. Patients with complete base width measurements (n = 32) showed slightly lower mean BMI (24.0 vs. 26.1 kg/m^2^) and similar age (55.0 vs. 51.4 years) compared to those with incomplete data (n = 18); the incomplete group included a higher proportion of reduction/mastopexy candidates (72% vs. 41%), suggesting that findings may underrepresent this indication and higher-BMI patients. Formal missing data analysis was not performed, and the degree to which incomplete records may have introduced systematic bias remains unknown. This limitation should be weighed when interpreting the reported associations.
- Formal inter- and intra-observer reliability was not assessed, as the measurements were collected during routine clinical practice and not within a prospectively designed and standardized research protocol. Measurement uncertainty may therefore be higher for parameters with gradual anatomical boundaries, particularly in patients with higher BMI, which may introduce systematic bias in the direction of the observed associations.

Despite these limitations, the study has strengths: it uses standardized clinical measurements, addresses an understudied proportional question (vertical vs. horizontal scaling), and explores a link between anthropometric measurements and clinical practice that may inform future preoperative assessment frameworks.

### 4.4. Future Directions

Future prospective studies incorporating parity, breastfeeding history, hormonal status, and longitudinal weight changes are required to further disentangle biological effects from measurement-dependent footprint appearance. Validation in larger, multicenter cohorts with standardized anthropometry would strengthen generalizability. Prospective longitudinal designs following patients through weight change could help determine whether the observed BMI–shape relationship is causal and reversible.

Combining simple linear indices such as the H/B ratio with three-dimensional volumetric metrics and objective measures of thoracic skeletal width (e.g., chest wall dimensions on imaging) may provide a more comprehensive characterization of breast footprint morphology. In addition, biomechanical modeling integrating tissue mechanical properties, fascial attachments, and gravitational effects could help explain why apparent base width preferentially expands and support more individualized surgical planning. Finally, evaluating whether the H/B ratio is associated with patient-reported outcomes after augmentation, reduction, or mastopexy would be necessary to assess its potential clinical relevance.

## 5. Conclusions

The H/B ratio represents a simple, surface-based descriptor of apparent breast footprint proportions derived from routine clinical measurements. In this exploratory retrospective cohort, higher BMI was associated with greater horizontal breast width, whereas vertical footprint height showed less pronounced variation, resulting in flatter apparent proportions with increasing body mass. These observations likely reflect BMI-related soft-tissue redistribution influencing clinically assessed footprint dimensions. Given the exploratory design and limited sample size, the findings are hypothesis-generating and require prospective validation in larger cohorts before broader clinical application can be considered.

## Figures and Tables

**Table 1:** Descriptive statistics of study population (n = 50).

Variable	n	Mean	SD	Min	Max	95% CI
Height (cm)	50	164.20	5.37	151.00	180.00	162.71–165.69
Weight (kg)	50	66.82	14.20	48.00	110.00	62.88–70.76
BMI (kg/m^2^)	50	24.74	4.78	18.22	39.90	23.41–26.06
Band size (cm)	50	81.60	7.45	70.00	100.00	79.53–83.67
SSN–N (cm)	50	27.39	5.77	18.00	42.50	25.79–28.98
N–IMF (cm)	46	9.67	3.35	3.50	18.50	8.71–10.64
Breast base width (cm)	32	14.32	3.30	10.00	24.50	13.18–15.46
Breast height (cm)	30	11.26	1.34	8.00	13.50	10.78–11.74
H/B ratio	29	0.83	0.16	0.47	1.10	0.77–0.89
Inter-nipple distance (cm)	41	22.23	4.98	14.50	38.00	20.71–23.76

Note: Sample sizes (n) vary across variables because some measurements were not documented in the original clinical records. Due to retrospective anonymization, missing values could not be recovered.

**Table 2:** Distribution of planned surgical procedures.

Planned Procedure	n	Percentage
Reduction/Lift	26	52.0%
Implant Exchange	16	32.0%
Augmentation	6	12.0%
Lipofilling	2	4.0%

Note: Values represent the indication for surgery as recorded.

**Table 3:** Bivariate associations between anthropometric predictors and breast morphology metrics.

Predictor	Outcome	n	Spearman ρ	*p*-Value	*q*-Value (FDR)
BMI	Base width	32	0.691	<0.001	<0.001
BMI	Height	30	0.327	0.0777	0.091
BMI	H/B ratio	29	−0.414	0.0255	0.032
Band size	Base width	32	0.621	<0.001	<0.001
Band size	Height	30	0.070	0.714	0.714
Band size	H/B ratio	29	−0.549	0.0021	0.004

Note: Spearman rank correlation coefficients (ρ) are presented alongside raw *p*-values and Benjamini–Hochberg FDR-adjusted *q*-values to account for multiple testing. Significance was defined as *q* < 0.05 [14].

**Table 4:** Multivariable OLS regression models predicting breast dimensions from BMI and thoracic width.

Outcome	n	β (BMI)	95% CI (BMI)	*p*-Value	β (Band Size)	95% CI (Band Size)	*p*-Value	Adjusted R^2^
H/B ratio	29	−0.046	[−0.103, 0.011]	0.108	−0.056	[−0.113, 0.001]	0.053	0.256
Breast Base width (cm)	32	1.820	[0.892, 2.748]	<0.001	0.948	[0.020, 1.876]	0.046	0.513
Breast Height (cm)	30	0.484	[−0.057, 1.025]	0.0777	−0.142	[−0.683, 0.399]	0.595	0.047

Note: Coefficients (β) are semi-standardized, representing the change in outcome (cm or ratio) per 1-SD increase in the predictor. Base width (cm) and height (cm) refer to absolute breast dimensions. Adjusted R^2^ indicates the proportion of variance explained by the combined model. *p*-values adjust for the simultaneous presence of both predictors. Variance inflation factors were 1.20, 1.24, and 1.23 for the H/B ratio, base width, and height models, respectively; values were identical for both predictors within each model. Condition numbers of the mean-centered and scaled predictor matrices ranged from 1.55 to 1.61. Both metrics indicate negligible multicollinearity.

## Data Availability

The anonymized raw data supporting the findings of this study and the analysis scripts are available from the corresponding author upon request.

## Abbreviations

The following abbreviations are used in this manuscript:

BMI	Body Mass Index
CI	Confidence Interval
FDR	False Discovery Rate
H/B	Height-to-Base-Width Ratio
IND	Inter-Nipple Distance
N–IMF	Nipple–to–Inframammary Fold Distance
OLS	Ordinary Least Squares
SD	Standard Deviation
SSN–N	Suprasternal Notch–to–Nipple Distance
ρ	Spearman Rank Correlation Coefficient
